# The Cat Is Already Out of the Bag: Humane and Pragmatic Solutions for Cats on Dairy Farms. Reply to Calver et al. It’s Premature to Encourage Working Cats for Rodent Control on Australian Dairy Farms

**DOI:** 10.3390/ani16030438

**Published:** 2026-01-30

**Authors:** Kate Dutton-Regester, Jacquie Rand, Vanessa Rohlf, Pauleen Bennett, Rebekah Scotney

**Affiliations:** 1 Australian Pet Welfare Foundation, Kenmore, QLD 4069, Australia; research@petwelfare.org.au; 2 School of Veterinary Science, The University of Queensland, Gatton, QLD 4343, Australia; rebekah.scotney@uq.edu.au; 3 School of Psychology and Public Health, La Trobe University, Bendigo, VIC 3552, Australia; v.rohlf@latrobe.edu.au (V.R.); pauleen.bennett@latrobe.edu.au (P.B.)

**Keywords:** animal welfare, farm cats, rodent management, One Welfare, farmer wellbeing, food safety, sterilization, working animals, barn-cat programs

## Abstract

For millennia, cats have been valued worldwide as biological agents for rodent control. Our previous qualitative research found that Australian dairy farmers valued cats for rodent management and companionship, while also highlighting welfare and operational challenges when populations were unmanaged. We therefore argued for a structured, humane management approach. Critics questioned our methodology and portrayed our publications as a blanket endorsement of placing cats on farms. Here, we clarify the scope and limitations of our earlier work and reaffirm that unmanaged cats can create significant risks, including disease transmission, poor welfare, environmental concerns, and psychological stress for farmers and veterinary professionals tasked with lethal control. Responsible management, through sterilization, feeding, healthcare, and formal recognition of some cats as working animals, has the potential to reduce these harms while aligning with farmer values and food safety requirements. Farmers also supported barn/working-cat programs to replace sterilized cats lost through attrition and because they recognized the wellbeing impact on shelter staff required to humanely kill healthy cats. While more research is needed to empirically examine the benefits of the humane management of farm cats, alternatives to cats suggested by critics, such as owls or dogs, lack equivalent evidence or feasibility in dairy systems. Given that cats already exist on many farms, we conclude that responsible management offers a pragmatic, humane, and One Welfare-aligned pathway while longer-term studies are undertaken.

## Simple Summary

Cats have lived on Australian dairy farms for generations, where farmers often value them for helping control rodents and for companionship. However, when cat populations are unmanaged, problems can arise, including poor welfare, disease risks, environmental impacts, and distress for farmers who may feel forced to use lethal control methods. In this article, we respond to critics of our earlier work by clarifying that the cats already present need to be cared for in a structured and humane way. Responsible management involves sterilization to prevent uncontrolled breeding, providing food and healthcare, and recognizing some cats as working animals. These measures can decrease the risk of zoonotic disease and wildlife predation, improve animal and human wellbeing, and ease the emotional burden on farmers and veterinarians involved in lethal management. Farmers in our study supported recognizing cats as working animals so costs of care could be tax-deductible. They also supported barn/working-cat programs to replace sterilized cats lost through natural attrition and because they recognized the impact on the wellbeing of shelter staff who would otherwise be required to humanely kill timid and fearful cats. While we agree wholeheartedly that more research is needed to assess long-term outcomes and the minimum effective number of cats for rodent control, waiting for perfect evidence would allow current problems to continue. Our message is simple: managing the cats already living on farms is a practical, humane approach that benefits animals, people, the environment, and farming systems.

## 1. Introduction

For thousands of years, cats have been valued worldwide as highly effective controllers of rodents, a role documented across ancient, medieval, and modern societies [1,2]. In Australia, cats have been viewed as an asset for rodent control especially around stored grain and were purposely introduced onto rural properties to control mice and rabbits [3]. Many dairy farmers use cats for rodent control, often in combination with rodenticides [4].

Our two recent papers [5,6] explored the views of dairy farmers regarding the value and challenges of having cats on their property. This qualitative research involved an exploratory study using semi-structured interviews of 15 farmers from 9 of the 10 dairy farms participating in two free cat sterilization programs operated by the Australian Pet Welfare Foundation in the city of Ipswich, Queensland, and the Animal Welfare League NSW in the Bega Valley Shire, NSW, Australia.

Key findings from the interviews were that these dairy farmers valued cats for their contribution to rodent management (“we couldn’t do without them, otherwise you’d be overrun with rats”) and the subsequent cost savings from rodent damage (“cats are cheaper than an electrician bill”). They regarded them as more effective than poisons (“you would still see rats and mice even when you had baits—there’s absolutely nothing as efficient as a dozen cats”) and also less hazardous than poisons (“we don’t like the baits around because of our dogs and little children”). They highlighted that unmanaged cat populations were associated with unwanted behaviors such as fighting and poor cat welfare (“every time a cat was on heat, they would mate and all the boys would fight”), as well as environmental risks (“they used to pee all over everything in the shed”). Farmers advocated for the recognition of some cats as working animals to facilitate their responsible management through sterilization, vaccination, feeding, and basic healthcare (“we need cats around that area [dairy] and around the hay shed to do what they need to do. So I think if you’re classed as a prime producer, you should have the full right to be able to claim cat deductions)”. They also often viewed them as companions in the dairy, as well as pest managers (“we enjoy their company at the dairy—someone to have a chat to when you’re there by yourself”).

Our findings were not intended to be generalized to all dairy farms nor to imply a population-wide prevalence of particular practices or views. As with all voluntary qualitative research, our sample is subject to selection and social desirability bias, and the views reported reflect the perceptions and lived experience of participating farmers rather than population-wide data. Importantly, we did not advocate introducing cats onto farms where they do not currently exist. Our focus was on reporting the views of these dairy farmers regarding cats that already resided on their dairy properties, especially the impact of the sterilization program.

Farmers stated that lethal methods are distressing and therefore not used efficiently to control the population, hence the need for humane, cost-effective strategies for cat management that also meet food safety audit requirements. Farmers expressed concern about the costs for sterilization (“the cost is too great to have to get all those cats done ourselves”) and supported that the cats’ care be tax-deductible: “Poisons are a tax deduction, so why can’t the cats be”. It was also stated that “they’re definitely doing a job, and it’s probably more important than some working dogs”, noting that working dogs and horses are tax-deductible but cats are not. This reasoning is consistent with existing regulatory practice in Australia, where working dogs are recognized in law and taxation frameworks because of their functional role on farms, without the prior requirement for the type of controlled trials being requested by Calver et al. for cats [7,8,9,10]. The United States of America (USA) and the United Kingdom (UK) recognize the role of cats in rodent control and permit them to be classified as working animals, making their care tax-deductible [11,12,13].

Farmers also were concerned about natural attrition if all cats were sterilized, and hence supported the idea of barn-cat programs [14] “where the farmers can take these ready-made cats that are sterilized, vaccinated and everything’s been done for them, and they’re ready to go into a working program”. They also commented that obtaining sterilized replacement cats from shelters is “a great idea to save euthanizing the cats”.

Calver et al. [15] characterize this as an “enthusiastic endorsement” of farm cats for rodent control and argue it is premature in the absence of controlled trials. Our aim is not to enthusiastically endorse cats as the single or ultimate solution to rodent control but to acknowledge that across cultures and through centuries, cats have been consistently used as the primary natural predator for controlling rodent pests in agricultural and stored-grain systems [1] and are already present on many Australian farms, including dairy farms. They are perceived as valuable for rodent control (“we couldn’t do without them, otherwise you’d be overrun with rats”). We argue they should be humanely managed because of the negative impacts of uncontrolled numbers of cats.

While we agree that more qualitative and quantitative data are sorely needed, Calver et al.’s portrayal downplays the practical and ethical realities facing farmers, veterinary professionals, and communities. The issue is not whether cats should be introduced onto dairy farms—they are already there in significant numbers—but whether they remain unmanaged, with all the welfare, zoonotic, and human wellbeing risks that entails, or could be managed effectively through structured, financially supported, evidence-informed programs. Humane, structured management reduces the problems associated with unmanaged populations and allows cats to play an integrated role within wider pest management systems, consistent with One Welfare principles.

In this response, we do enthusiastically endorse Calver et al.’s [15] call for more research, particularly long-term studies that may take many years and cost many thousands of dollars. In the interim, we address the concerns raised by Calver et al. [15] and clarify why the responsible management of dairy farm cats is a pragmatic, humane, and necessary step forward.

## 2. Cats and Rodent Control

The dairy farmers in our study consistently identified cats as part of their rodent management toolkit, particularly in the context of food safety audits. Rodent control is not optional but a regulatory requirement: Australian dairy farms must implement pest management plans that prevent the harborage of pests, including rodents, under Standard 3.2.2 of the Australia New Zealand Food Standards Code [16]. This regulatory pressure shapes farmer choices, but it does not prescribe specific methods. Farmers in our study reported that auditors accepted cats as part of compliance strategies, and they consistently expressed a preference for cats over poisons, which they viewed as hazardous to dogs, children, and wildlife (“baiting’s not great for the other wildlife, and we’ve got dogs”; “I’d prefer not to use the baits”). They also viewed rodenticides as more costly and less effective than cats: “The baits weren’t doing the job, not like cats,” and “the cats seem to work everyday where baits are only ever any good while you’ve got bait out”. The presence of cats reduced rodenticide use: “We haven’t used rodent baits for probably 4 years now, since we’ve had cats”. Cats were also valued for preventing equipment damage and breakdowns in the dairy, which were costly and time consuming to repair and disruptive to dairy operations: “you can’t have breakdowns in the dairy—it’s gotta go twice a day, every day”. The farmers consistently remarked on their perception that the cats were very effective in preventing equipment damage (“we haven’t had another machine eaten [by mice] since the cats have been here”) and stated this resulted in cost saving on repairs and on baiting, commenting “I would say they were saving us between two and three thousand dollars a year, at least,” and “we need some cats at the dairy because we just kept having problems with our wiring, cause the rats keep getting in and chewing them.”

These perceptions may or may not be representative of farmers more broadly, or even dairy farmers more broadly, as is inherent in qualitative sampling, but they are critical, because behavioral theory shows that risk perceptions and perceived benefits strongly influence which management practices people are willing and able to adopt [17,18,19]. As Calver et al. [15] acknowledge, using qualitative methods to deeply explore the lived experiences of a small and selective sample is a legitimate means of starting a conversation about a topic and of directing researchers to areas requiring more extensive investigation. Indeed, our exploratory qualitative research has highlighted a number of research hypotheses and questions that need substantial funding to test with farm studies, given the urgent need for data to assist policy making.

Calver et al. [15] cite Elton 1953 [20] to argue that cats are not effective. Yet Elton’s work, while valuable historically, was observational, limited to a handful (*n* = 6) of farms and a cottage, and lacked the rigor expected in modern ecological research. Importantly, even Elton [20] concluded that cats could be effective at maintaining low rodent densities once infestations were suppressed and when several cats were supported near farm buildings. This aligns with farmer testimony: cats are not a silver bullet, but they provide a maintenance role within integrated pest management systems.

Concerns about cats attracting rodents through uneaten food can be addressed through feeding protocols. Best practice, applied in managed cat colonies globally, involves feeding nutritionally appropriate cat food sufficient to meet energy requirements. Typically, this involves providing food twice daily for 20–30 min and removing any leftovers [21,22]. This approach meets the cats’ energy and nutritional needs, reduces predation pressure on wildlife [23], and prevents food waste from attracting rodents. It has the added benefit of facilitating monitoring cat health at feeding times.

Calver et al. [15] also cite more recent studies suggesting variable outcomes. We acknowledge this variability [24], but it does not mean cats are ineffective. Rather, it suggests that outcomes depend on context, species, and numbers. For example, Wijburg et al. [25] utilized a baited camera-trapping survey of 758 private gardens in the Netherlands from 2016 to 2023 (31  ±  12.2 survey days per camera) to measure mammal presence and showed that cats were a negative predictor for the presence of rats (*p*  =  0.004) and mice (*p*  =  0.018). Mahlaba et al. [26] conducted a survey of rodent activity at 40 rural homesteads in central Swaziland and found that the presence of cats and dogs together significantly reduced rodent activity (β = −1.10) and foraging (β = −1.32). Hillar et al. [27] assessed rodent feeding activity in eight poultry farms in Argentina and demonstrated that cat fur odor reduced the probability of food consumption by rodents (*p* = 0.0078), indicating that even deterrence effects can contribute to management. By contrast, a field study of the feeding behavior of wild Norway rats at a farm in Poland found that exposure to predator odors (including cats and dogs) did not induce predator avoidance behaviors in rats [28]. But this may reflect the need for higher cat density or different ecological contexts. Taken together, these findings highlight that context matters. Interestingly, rat body size differs markedly across environments: rats in rural areas are reported to be smaller than those in urban areas, so cats in rural settings may be better able to control rat populations in that they may be able to kill all age classes of rats, including those of breeding age [29,30]. In contrast, because of the larger size of urban rats, cats in cities are less likely to prey on adult rats than juvenile and subadult rats [29,30].

However, it cannot be ignored that throughout human history and across multiple continents, cats have been the most widely relied-upon biological control for mice and rats in food stores, farms, homes, and ships [31,32]. In Australian rural culture, the use of farm cats as vermin controllers is long-standing, even if formal rodent-control data are limited [33].

The more productive path is as follows: first, to examine how cats can complement existing rodent-control methods on dairy farms, not to dismiss them entirely; second, to explore how the effective management of existing cats can mitigate known environmental risks associated with unmanaged cats; and third, to establish recommendations for minimum effective numbers for varying types of farming operations. Integrated pest management recognizes that different methods serve different functions. Cats provide continuous pressure on rodent populations near farm infrastructure [26,27], reducing the need for repeated poison use and supporting food safety compliance. Where studies suggest a limited effect, the more relevant question is whether cat numbers were optimal or whether additional measures were required. Further prospective research is needed to determine the most effective numbers of cats for different farm contexts, as well as the costs and benefits, measured with a One Welfare lens, of various combinations of methods for increasing food safety. For example, combinations of increased investment in rodent-proof grain and crop storage, rodenticide use, and working cats must be examined. In the meantime, the management challenge remains: the cats are already there, and the focus should be on managing them humanely and effectively.

## 3. Cats and Risks of Zoonotic Disease and to Wildlife

Calver et al.’s [15] rebuttal highlights risks to wildlife. However, they ignore the alternative impact of rodenticide use on wildlife [34,35,36,37], the potential for human poisoning, especially in children [38,39], and the need for increasingly more potent rodenticides. Recent genetic screening of urban rat populations in Australia found a high frequency (≈54%) of a Vkorc1 mutation associated with resistance to second-generation anticoagulant rodenticides (SGARs), indicating increasing potential for rodenticide failure [40].

They also do not address the effects of uncontrolled populations of rats on wildlife, particularly native species of birds (especially eggs and chicks of ground-nesting species), small mammals, including bats, and other small vertebrates (e.g., frogs and lizards) and invertebrates [41,42]. One study in remnant bushland in Sydney demonstrated the positive impact cats had by reducing the numbers of rats that predate bird nests [43]. In a study involving the placement of 20 artificial birds’ nests with eggs in each of Sydney’s 24 metropolitan bushland sites, it was found that the higher the cat activity (estimated by the amount of cat feces), the less nest raiding that occurred [33,43,44]. The authors concluded that cats reduced nest raiding by suppressing the activity of nest raiders, such as introduced black rats. Finally, context matters. Dairy farms are intensively modified landscapes with little or no native vegetation and limited habitat for native species. Extrapolations from bushland or suburban studies [45], contexts that differ markedly from intensively modified dairy landscapes, overstate risks in dairies, where rodent pressures are the dominant ecological concern.

We acknowledge Calver et al.’s [15] concerns relating to both wildlife and zoonotic disease from farm cats but stress that unmanaged cats pose the greatest risks. Responsible management substantially reduces them. Younger cats hunt more and are associated with greater zoonotic disease risk than older cats [46]. *Toxoplasma gondii* oocysts are shed almost exclusively by young cats during their primary infection, with re-shedding by adults rare [47,48]. An intervention study of three pig farms in the Netherlands demonstrated that removing kittens and preventing kitten births through sterilization reduced *T. gondii* seroprevalence in pigs by 67% within 12 months by reducing environmental contamination and lowering infection risk in pork [48]. By sterilizing farm cats, the group at highest risk of shedding is eliminated from the population, directly addressing a key zoonotic hazard. In contrast, unmanaged breeding populations will continuously produce susceptible kittens, perpetuating risk.

In addition, feeding cats nutritionally balanced food that meets their energy requirements eliminates the need to consume prey for survival, further breaking the infection cycle [23,49]. A study of scat analysis of pet cats from rural southern Chile found that vertebrate remains were 4.7 times more likely to occur in the scat of poorly fed cats than that of adequately fed cats (*p* < 0.01) [49]. In the same way, appropriate feeding of farm cats prevents infection with sarcocystosis; uncooked meat and offal should never be fed to cats (or dogs), and livestock carcasses in the vicinity of farm buildings should be promptly removed [50].

Calver et al. [15] cite estimates that cat-associated pathogens cost Australia billions of dollars annually [45]. We do not dispute the burden of disease, but unmanaged, uncontrolled breeding is precisely what sustains these risks. Our proposal to manage existing farm cat populations through supported sterilizing, vaccination, feeding, and parasite control is entirely consistent with the call to minimize cat densities and disease risk on dairy farms. The difference is in approach: rather than attempting to eliminate cats through lethal measures—which experience shows is costly, traumatic, often ineffective, and unlikely to be supported by farmers [51,52,53,54,55,56,57,58]—our framework has the potential to stabilize and reduce populations over time to minimum effective numbers while improving welfare outcomes and decreasing zoonotic and wildlife risks.

Calver et al. [15] also argue that our logic is contradictory: if well-fed cats hunt less, then they cannot control rodents. Our reading of the evidence is that cats contribute to rodent control in two ways: direct predation and deterrence. Multiple studies show that rodents alter their foraging behavior in the presence of cats and dogs, creating a “landscape of fear” effect even when kills are few [25,26]. Experimental trials confirm that cat fur odors reduce rodent food consumption in poultry farms [27]. While Stryjek et al. [28] found that Norway rats foraging in barns within their established home ranges did not avoid predator odors when food was abundant, this highlights that effectiveness is context-dependent, not absent.

Although well-fed cats still hunt opportunistically [49], feeding nutritionally balanced food to meet the cats’ energy requirement removes the necessity to predate for survival. In an intervention study of pet cats in England, feeding cats (*n* = 66) nutritionally balanced food that met their energy requirements reduced the number of animals the cats killed by 36% (*p* < 0.001) [23]. In addition, sterilizing cats reduces their activity levels and energy requirements, as reproducing female cats require a 2–3 times higher energy intake than a sterilized cat [59,60,61,62].

Feeding cats adequately helps maintain healthy, stable cat numbers that exert consistent local pressure. Farmers in our study all fed their cats, and they reported that after sterilization, the cats were more likely to remain close to the dairy, appear healthier, and provide more reliable day-to-day suppression of rodents. As one farmer commented, “they used to bring birds in the office and there’d be feathers everywhere. We’ve noticed there’s none of that now. They don’t seem to be roaming as they used to.” Another explained before they were sterilized, “you’d see them [the cats] down in the gully or in the paddocks and they’d be out hunting, but now, I swear they just kind of hang around the dairy area”. Of course, these observations require empirical validation beyond what was possible within our initial study. In the absence of evidence to the contrary, however, it seems reasonable to assume that starving cats may disperse more widely, increasing wildlife predation and disease spread.

Calver et al. [15] further suggest that cats could vector emerging diseases, such as avian influenza, if fed unpasteurized milk from infected cows. This is a theoretical risk, but again, management is the distinction. In Australia, outbreaks of H7 high-pathogenicity avian influenza (HPAI) have occurred recently in poultry in Victoria, New South Wales, and the ACT, though they have since been eradicated, and Australia remains free of the globally circulating H5 strain [63]. Avian influenza is fundamentally a disease of birds, but spillover into mammals has been documented overseas, including cats, dogs, pigs, mink, seals, and dairy cattle [64,65]. Airborne mammal-to-mammal transmission of influenza A viruses has also been demonstrated experimentally in ferrets [66], reinforcing that spillover is possible. However, such risks are contingent on exposure routes. Managed farm cats should be fed balanced feline diets in appropriate amounts, ideally twice daily in monitored sessions, with leftovers removed. In regions where avian influenza is recognized, feeding of raw milk or poultry offal to any animals would be strongly discouraged. Health monitoring during feeding also facilitates early detection of illness. In contrast, unmanaged cats, scavenging opportunistically, are far more likely to encounter and spread pathogens, underscoring why structured management is essential [67,68,69]. A One Welfare lens demands proportionality and risk minimization: unmanaged cats perpetuate both animal and human harms, while managed cats reduce risk of zoonotic disease and of wildlife predation and improve cat welfare and human wellbeing while safeguarding livestock and food safety.

## 4. Welfare of Cats and People

The welfare of both cats and people is central to our argument, yet the human dimension is almost entirely absent from Calver et al.’s [15] critique. Unmanaged cat populations often suffer from poor health, hunger, untreated disease, and high mortality. This also negatively affects farmers’ job satisfaction, with one remarking “you feel shit when you look at a sick cat. It’s like, your mood changes when you see a sick animal”. In turn, farmers confronted with rising numbers have historically resorted to lethal methods such as shooting and blunt trauma or drowning kittens but commented “the shooting part was pretty stressful on the cat and on me” and “then you put it off for the next day, and the next day”. These actions are not only inhumane for the animals but also decrease job satisfaction (“it is not a job I enjoy”) and have the potential to cause significant moral injury to farmers, who in our interviews described distress and discomfort at being forced into such practices and therefore did not effectively control cat numbers. Again, we acknowledge that our qualitative methodology means that we cannot generalize from the farmers in our study to the wider farming community. However, it seems quite reasonable to assume that most farmers would dislike killing cats and kittens and prefer an alternative method of control if available.

Calver et al. [15] raised concerns that up to a quarter of cats were humanely killed. However, that only occurred in the initial phase of sterilization programs, when unmanaged populations were in excess and contained large numbers of sick cats. We strongly recommend that farm cat populations are managed in a timely way by sterilization and adoption of kittens to prevent excess and sick cats having to be humanely killed. Importantly, farms with the highest initial cat numbers had the highest number and proportion of cats euthanized, reinforcing the importance of early intervention before populations escalate. Timely intervention avoids the adverse effects on animal and human wellbeing. The burden of mass culling falls on farmers, veterinarians, veterinary nurses, shelter staff, animal management officers, and community carers, all of whom are repeatedly tasked with destroying healthy and treatable animals. Research in Australia and internationally shows that such practices create moral injury, compassion fatigue, and burnout among professionals [55,57,58,70,71,72]. More recently, qualitative research at the Port of Newcastle documented how lethal cat management inflicted trauma and grief on cat caregivers and psychological distress, resulting in signs consistent with post-traumatic stress disorder [57].

Responsible management—through sterilization, adoption pathways for kittens, feeding, vaccination, and parasite treatment—directly addresses these issues. Given that euthanasia rates were initially inflated on farms where initial cat numbers were uncontrolled and exceeded 20 cats, it is recommended that management by sterilization be implemented before these numbers are exceeded. Farmers in our study expressed strong support for these cat-caring measures, highlighting that they aligned with their values and reduced the emotional burden of lethal control. Recognition of some cats as working animals would formalize these responsibilities. Just as working dogs are entitled to appropriate care, so too should working cats be afforded food, veterinary attention, and oversight. Such recognition would also ensure consistency across species, making explicit that animals contributing to farm operations, and accepted as supporting food safety through rodent control, are treated with dignity and their welfare optimized.

Allowing costs of veterinary care and food for working cats to be tax-deductible would facilitate responsible cat management and minimize environmental risks associated with excessive numbers of cats. The cost of sterilizing the initial number of cats (median = 44) would be expected to range from A$10,000 to A$17,000, excluding vaccination, parasite control, and other healthcare costs. This is prohibitive, and as stated by one farmer, they do not spend money if it is “not putting money in vat”. Farmers also voiced that both cats and rodenticides were considered by food safety authorities as effective tools for rodent control, but only rodenticides are tax-deductible.

In sum, Calver et al. [15] mention welfare concerns only superficially, including mentioning concern about rodent welfare and focusing on animal numbers, but fail to account for potentially wider human impacts of lethal control. Humane, structured management should, in theory at least, reduce the need for mass killing, alleviate psychological stress in the veterinary and farming professions, and build more constructive relationships between farmers, regulators, and communities. While we agree that extensive empirical research is required to test these claims, a One Welfare approach requires that these human costs are considered alongside animal and environmental outcomes.

## 5. Numbers and Economics

Calver et al.’s rebuttal [15] questions how many cats are required per farm and whether they are economically viable. We acknowledged in our original papers that further research is required to quantify the role of cats in rodent management on Australian dairies [5,6]. Calver et al. [15] are correct that the optimal number of cats per property has not been determined, and we agree this should be a focus of future prospective studies. Farmers in our studies reported benefits across a wide range of numbers, typically between 10 and 30 animals depending on farm size, building layout, and rodent pressures. Where higher numbers were reported, these were also associated with poorer welfare outcomes and increased euthanasia because cats were sick or in excess, reflecting long periods without management intervention—precisely why timely management is required to stabilize populations. This also highlights why research is urgently needed to determine the most cost-effective methods of rodent control that optimize human and animal welfare, as well as environmental wellbeing. Industry bodies and state and federal governments need to support funding for research to determine the optimum combination of methods to maximize food safety for different farming contexts, namely, increasing the security of food storage, rodenticides, and working cats. For example, putting a lid on the food vat in a piggery was effective in reducing toxoplasmosis infection in pigs [48]. However, in other situations, rodent-proofing agricultural infrastructure is technically challenging and often prohibitively expensive, as effective exclusion requires specialized construction materials, structural modification, and ongoing maintenance [73].

Calver et al. [15] contrast our farmer-reported numbers with “international standards” from European studies, where average cat numbers per farm are much lower. Such comparisons may be misleading without careful consideration of context. Australian dairy farms are typically larger, with more extensive infrastructure and multiple rodent entry points, compared with the small-scale European holdings cited. It is therefore unsurprising that Australian farmers report higher numbers. The key point is not that more cats are inherently better, but that unmanaged proliferation creates welfare and disease risks and that further research is needed to determine the minimum number required for effective maintenance of rodent control under Australian conditions.

Economic realities are also central to farmer decision making. Dairy farmers in our studies consistently perceived cats as more cost effective than rodenticides, but sterilization and healthcare costs were prohibitive for their optimum management. This is not surprising; the average non-discounted cost at a private veterinary practice to sterilize a female cat typically ranges from A$300 to A$500 or more depending on the veterinary practice and whether the cat is lactating or pregnant, with males being approximately half this cost [74]. A number of farms had over 50 cats and up to 172 cats. At an average price range of A$225 to A$375, assuming half were male and half female, sterilization costs could range from A$11,250 to A$18,750 for 50 cats and A$39,375 to A$65,625 for 175 cats, excluding vaccination, microchipping, parasite control, and other healthcare costs. Farmers also expressed concerns about the resources required to trap and transport cats for sterilization and commented, “as a small business owner, that you know is very, very busy running a dairy farm and growing crops, feeding cattle and feeding calves and all the stuff that goes with it, and it’s seven days a week. To have someone to do a program and to do all the heavy lifting as far as the sterilization goes, that was a brilliant way to go.” These perceptions are important because they influence the adoption of management practices. Recognition of some cats as working animals would allow tax deductibility of associated costs, just as for working dogs, thereby removing a key barrier to responsible care. Such support would also strengthen compliance with required pest-management outcomes under the Food Standards Code.

Importantly, current regulatory frameworks for pet cats are not designed for the realities of farms with large, resident populations of cats around farm buildings. For example, in New South Wales a pet cat must be registered (~A$69), and if older than four months a breeder permit (~A$91) is also required, even if the cat is sterilized at the time of registration. Excess cat permits would also be payable in many jurisdictions. For farms with dozens of cats, these costs would be prohibitive. While working dogs must also be registered, the registration fee is waived, acknowledging their functional role. A comparable category for working cats is therefore necessary. Simply enforcing existing pet-cat regulations on dairy farms would be financially unworkable and would not resolve the welfare, zoonotic, or rodent-control challenges posed by unmanaged populations.

Calver et al. [15] are correct that detailed cost–benefit modelling has not yet been undertaken. We emphasized this gap ourselves and identified it as a priority for future research. In the meantime, farmer perspectives provide a valuable, practice-based foundation for policy and management discussions. The evidence to date suggests that cats can be an affordable and farmer-supported component of integrated rodent management, which also is recognized by authorities responsible for monitoring food safety. However, they need to be managed responsibly to minimize risks. Delaying action until more data are available risks perpetuating uncontrolled populations, with ongoing risk to wildlife and welfare harms. Recognizing some cats as working animals provides a pragmatic framework for delivering immediate improvements while building the evidence-base for refined economic and ecological modelling.

## 6. Alternatives and Integrated Management

Calver et al. [15] suggest that other predators, such as barn owls or farm dogs, could substitute for cats in rodent control. While these ideas warrant consideration, the current evidence does not justify neglecting to manage effectively and humanely the cats that are already present on dairy farms for rodent control.

Barn owls have been studied most extensively in oil palm plantations in Southeast Asia. Large-scale trials demonstrated that owls can suppress rat damage when high nest-box densities are provided and rodenticide use is limited [75,76]. However, outcomes were variable, depending on rat species, prey abundance, nest-box provision, and seasonality. Even in these systems, owls were not a rapid knock-down tool: early reductions in rodent damage typically required initial baiting until owl populations were established because acceptable suppression of rodents was achieved only after owl numbers increased over time. The most effective outcomes occurred when owls were combined with other methods rather than being relied upon as a stand-alone solution [75]. Reviews emphasize that owls are best viewed as one tool in integrated pest management (IPM), not as a stand-alone solution [77,78]. Crucially, no studies have tested their effectiveness or practicality in Australian dairies, which differ substantially from oil palm monocultures in habitat structure, infrastructure, and prey availability.

Dogs are also mentioned as a potential alternative. Some studies suggest that the combined presence of dogs and cats reduces rodent activity. Mahlaba et al. [26] reported that rodents foraged less and showed reduced activity where both cats and dogs were present together. However, dogs alone were not effective suppressors, and their contribution was context-dependent. Evidence indicates that cats can act both as predators and deterrents of rodents. For example, they may exert strong selective pressures on rodents’ behavior and physiology, with a study showing that rats exposed to cat odors had significantly higher behavioral inhibition (*p* < 0.01) and significantly higher neuronal activity in several regions of the brain (*p* < 0.001) than unexposed rats [79]. Exposure to the odor of cat urine or fur induces innate defensive and avoidance behaviors in rodents, reducing feeding or exploration when cat scent is present [24]. In contrast, evidence of the impact of dogs is more equivocal: some studies report no avoidance by native rodents of dog feces (*p* = 0.20) [80], while others found feeding rates decreased significantly in response to dog integumentary odors (*p* = 0.037) [81]. One study showed that removal of dogs from the environment altered rodent behavior, typically increasing their activity and visibility, although the underlying mechanisms remain unclear [82]. The dogs observed in relevant studies were largely mixed-breed, free-ranging animals, and their deterrent effect was attributed to barking, scent-marking, or territorial activity rather than direct hunting [83,84].

In the Australian dairy farm context, working breeds such as kelpies and cattle dogs are essential for stock management but are typically excluded from milking and milk-handling areas to manage contamination risks, consistent with food safety requirements to prevent the unnecessary movement of animals that are non-essential to operations [85]. Traditional ratting breeds such as terriers have historically been used in supervised hunts to kill rats on farms, but this is not recognized as a feasible or humane substitute for continuous, farm-wide rodent control. While these hunts can remove individual rats, they are episodic, labor-intensive, and dependent on human supervision. Because rodents reproduce quickly and continually reinvade from surrounding areas, occasional hunting cannot deliver the ongoing suppression required for food safety compliance. Moreover, terrier hunting is inconsistent with modern pest management and animal welfare standards: it is not a continuous method, is difficult to monitor, and it raises practical risks in dairy settings [85]. In contrast, cats already live on many Australian dairies, and the farmers in our study perceived that they provided a constant, localized deterrent and predation presence around dairies, barns, and feed stores. When managed responsibly, they can contribute to integrated pest management in a way that is both more feasible and more compatible with current farming systems than sporadic dog-based hunting.

The key point is that both owls and dogs may contribute in some contexts, but neither provides a ready substitute for cats. Both also lack the robust, farm-specific trial data that Calver et al. [15] are requiring for cats. It is inconsistent to dismiss cats for insufficient quantitative data while advocating for untested measures that face their own significant ecological and welfare limitations. The critical distinction is that cats are already present on many Australian dairy farms. Farmers are managing them now—often through lethal means—because affordable, humane alternatives are lacking. Our argument is not to import new predators with potentially negative impacts on wildlife but to improve the welfare and effectiveness of the ones already there.

This debate should not be framed as “cats or owls” or “cats or dogs.” Integrated pest management recognizes that multiple tools are needed. Cats, when managed responsibly, can provide continuous, localized suppression of rodent activity near farm infrastructure. According to the farmers we interviewed, cats were perceived to reduce reliance on poisons and were accepted by food safety authorities responsible for food safety audits. Comments regarding the food safety audit included, “if you put baits out, you have to tell them when and where you put baits, but we literally write, we have cats, they’re our vermin control. That’s all we have to state and that classes for our food safe handling.” Owls may have a complementary role in landscapes where nest boxes are feasible, and dogs may contribute indirectly, in combination with cats, but the immediate and unavoidable management question is how to deal with the cats that are already present. Humane, structured management—sterilization, vaccination, feeding, and health oversight—has the potential to minimize risks and maximize benefits while providing a basis for future research on how other methods can be integrated into dairy systems.

## 7. Precautionary Principle and Research Needs

Calver et al. [15] invoke the precautionary principle to argue that working cats should not be recognized or managed until long-term, quantitative studies are complete. However, the precautionary principle states that lack of full scientific certainty should not be used as a reason to postpone taking action to prevent environmental or human health damage [86,87]. It advocates for a proactive approach, requiring preventive measures to be taken to prevent or minimize that harm rather than waiting for conclusive proof [86,87]. This principle is a guiding framework for public policy and environmental law. There is credible evidence that sterilizing cats improves their health and welfare in free-living domestic cat populations [88,89] (also noted by the farmers) and that when high sterilizing rates are achieved, together with adoption of kittens and prompt sterilization of immigrant cats, cat numbers decrease over time [53,88,90]. Therefore, although we agree with Calver et al. [15] that more research is needed, it seems inadvisable to do nothing until all evidence is perfect. Best practice often requires that we act in the face of uncertainty to prevent known harms. Unmanaged farm cat populations already present significant and well-documented problems: poor animal welfare, over-reliance on lethal control by farmers, zoonotic disease risks, and hazards to wildlife. Simply waiting for another decade of data would perpetuate these harms.

Our argument is that immediate, humane management will reduce those risks now, while research continues. This is consistent with the way precaution has been applied in other agricultural contexts. For example, sterilizing cats to decrease toxoplasmosis risk in piggeries has led to management programs that were implemented and budgeted despite data gaps [48]. Similarly, cat sterilizing programs, including long-term university campus studies in the Australia and the USA (e.g., [53,88,90]), have demonstrated that when resident cats are managed by sterilization and immigrant cats are promptly sterilized, cat numbers decline over time and overall cat health improves. These outcomes provide a strong, evidence-based rationale for implementing management now rather than maintaining the status quo.

We emphatically agree with Calver et al. [15] that more evidence is needed. Indeed, both of our original papers emphasized this, calling for prospective, multi-site, long-term studies to quantify rodent control, disease transmission, wildlife interactions, and economic costs and benefits. Nonetheless, we reject the suggestion that management should be paused in the meantime. Dairy farmers already have cats, they already face rodent-control obligations under food safety audits, and they already perceive cats as part of their management toolkit. The question is not whether cats should be introduced, but whether the cats that are already present will remain unmanaged or be brought under responsible stewardship.

Responsible management—sterilizing, vaccination, parasite control, feeding protocols, adoption of kittens, and monitoring—directly addresses the welfare, zoonotic, and environmental risks that Calver et al. [15] raise. It also creates the infrastructure for exactly the kind of rigorous research they are asking for by stabilizing cat numbers and providing baseline data for long-term monitoring.

In this context, precaution supports action, not paralysis. The evidence-base is sufficient to justify humane management now, while additional studies refine best practice. Doing nothing is not a neutral position—it perpetuates animal suffering, disease risk, and lethal control methods that harm both animals and people.

## 8. Barn- and Working-Cat Programs

Farmers voiced support for working/barn-cat programs whereby healthy cats that are timid or fearful of humans, and would otherwise be humanely killed in shelters, were rehomed as working cats [22,91,92]. In these programs, typically sterilized and vaccinated cats are provided to people seeking a working cat for their property, which are characteristically in highly modified environments where rats are attracted by food sources that cannot be readily rodent-proofed. This includes farms with stored animal feed or crops, horse barns, orchards, wineries, and factories [14,93].

The farmers explained that their support was because of a concern about the effect of natural attrition of sterilized cats over time and the need to maintain adequate numbers if all cats were sterilized. As explained by one farmer, “[if] there’s no replacement plan going on, what am I going to do when these cats eventually pass away?’ We won’t have a cat. We’ll have to go and buy some,” and “there should be some measure whereby we can replace these cats without huge expense”.

Another reason for their support of working-cat programs was “that’s a great idea because when we were looking for cats, we were struggling to find the first couple of cats that we needed”. Another stated that “for a program to be out there where the farmers can take these ready-made cats that are desexed, vaccinated and everything’s been done for them, and they’re ready to go into a working program, that’s a great idea.”

They also identified with the negative impact on the wellbeing of shelter staff who are tasked with killing healthy animals, saying “ I think that’s a great idea to save euthanising the cats.” Human wellbeing is rarely considered by conservationists when advocating for cat management, particularly when recommendations rely on lethal control. However, when humans have a relationship with the cats, lethal management damages human wellbeing. The dairy farmers in our study described the cats as companions, for example, “I enjoy having [the cats] around. When you get here at three o’clock in the morning, you’re on your own, we talk to them. They’re company, I guess”; “when they come in, they meow to you, and you go out and you feed them and give them a pat’; and “ Dad can be in a cranky mood and he’ll come in and be cranky at us, and then he’ll see the cats and just be talking to them like they were the best things in the world.” Similarly, they expressed dislike for having to cull them to manage numbers: “A tap on the head, I found that’s the quickest and easiest way [to kill the kittens]. It’s not the most pleasant way,” and “you don’t think about it too much because you don’t want to,”.

This support for working/barn-cat programs was not only about the need to replace cats over time if all were sterilized but also the farmers connected with the emotional burden on shelter workers who currently must kill healthy but timid cats that cannot be adopted. In Australia, approximately 33% of cats entering shelters and municipal facilities are humanely killed, mostly because they are timid and fearful of unfamiliar people and environments and are deemed unadoptable [94,95]. Research consistently shows that euthanizing healthy or treatable animals has profound psychological impacts on shelter workers, including moral injury, compassion fatigue, and elevated risk of burnout and suicide [96,97]. Shelter staff describe the need to humanely kill unadoptable but otherwise healthy animals as one of the most distressing aspects of their role, leading to chronic stress, reduced job satisfaction, and symptoms consistent with post-traumatic stress [58,98]. These impacts can accumulate over time, contributing to high turnover and diminished wellbeing across the animal-care workforce [71,96,98,99]. This reinforces the need for humane alternatives such as working-cat and barn-cat programs, which the farmers supported. Recognizing working cats within structured, humane programs may therefore reduce unnecessary euthanasia, improve human wellbeing, and meet the ethical expectations increasingly required in contemporary agricultural and animal-care policy.

## 9. Conclusions

We agree with many of the points raised by Calver et al. [15] but conclude that the debate should not be about whether cats should be on dairy farms. They are already there. The real question is whether they remain unmanaged, with all the associated welfare, zoonotic, wildlife, and human wellbeing risks, or whether they could be managed responsibly and humanely as working animals. Our original study documented dairy farmer perspectives and proposed practical solutions: sterilization, vaccination, feeding, and basic healthcare. These measures were perceived to improve animal and human welfare, reduce disease and wildlife predation, and support farmer wellbeing. They are consistent with One Welfare principles, recognizing the interconnected wellbeing of animals, humans, and the environment. Future research is essential to validate and extend the understanding we acquired through exploring in-depth the lived experiences of our sample of dairy farmers, but action cannot wait. Recognizing some cats as working animals would provide a pragmatic framework for delivering immediate benefits while building an evidence-base to better inform future policy.

## Abbreviations

The following abbreviation is used in this manuscript:

IPM	Integrated Pest Management
